# Case of Colica Pictonum, from the Medical Employment of Acetate of Lead

**Published:** 1852-04

**Authors:** L. S. Joynes

**Affiliations:** Accomack


*Case of Colica Pictonum, from the medical employment of Acetate of Lead.* With remarks. By L. S. Joynes, M. D., of Accomack.—The following case is communicated, with the hope that it may prove a useful caution to some of the younger members of the profession, who may be inclined, from over-confidence in the assurances of many of our standard authors, to make too free a use of the potent drug above mentioned. It cannot be doubted that young practitioners in general are too prone to adopt *heroic* methods of practice, and to esteem boldness and decision the chief qualities of a successful physician. Of all the rich fruits which experience brings with it, not the least valuable, certainly, is *caution* in the employment of active remedial agents—all of which, without exception, are potent for evil as well as for good.

It has often appeared to me, that one of the most valuable contributions that could be made to the science of medicine, would be a faithful record of the *real experience* of the profession in the use of the class of remedies just referred to—setting forth the deleterious effects which may be caused by antimony, opium, mercury, lead, &c., as prominently as the good they are capable of accomplishing. Such a record would furnish the most useful lesson of caution in practice that could be given. A desire to contribute to this useful kind of information has influenced me in furnishing the following case for publication—in regard to which, I must admit that the length of time which has elapsed since its occurrence renders my history of it less complete than is desirable, inasmuch as I made no record of it at the time, and am compelled to rely chiefly on memory for the details.

On the 15th of October 1843, Mr. J. J. B., merchant, aged 25, consulted me on account of a chronic diarrhea, which had troubled him from time to time for several years, and which had always been obstinate and intractable. He had been treated by different physicians, who had prescribed a great variety of remedies, nearly all of which seemed of little efficacy. After listening to the details of his case, I became satisfied that acetate of lead and opium would prove the most efficient remedy; and as none of his previous medical advisers had prescribed this combination, I determined to give it a trial. I believe I had never before prescribed the acetate of lead, but I relied, very naturally, on the assurances, everywhere to be met with in the books, that the use of the drug is free from risk, provided a perfectly pure article be employed, and acetic acid in some form be prescribed in conjunction with it.

I accordingly selected the most perfect crystals of the acetate; added more than enough of distilled vinegar to neutralize any of the carbonate which might be present as an impurity; then adding the opium, I made the mass into pills. I also directed the patient, immediately after taking each pill, to swallow a teaspoonful of vinegar. The precise formula which I employed I am unable now to state, but my recollection is distinct, that the entire quantity taken was 30 grains, in the course of about four days. The medicine proved most efficient in the relief of diarrhea, the discharges being arrested more promptly than by any other remedy, and without any *immediate* ill effects. The patient and myself were both congratulating ourselves upon the favorable result, when one day, perhaps a week after he discontinued the pills, he complained to me of a pain in the epigastrium, radiating to the spine. As the patient was then apparently regaining his health, and in good spirits, I paid little attention to this complaint, supposing it to be a momentary gastralgic affection, occasioned by some imprudence in diet. Not long after, I learned that he was laboring under a severe attack of colic, and I was called on by Dr. Young of this place, his ordinary medical attendant, who came on purpose to ascertain the composition of the pills which the patient had been taking. The peculiar character of the colic with which he was at that time suffering, so strongly resembled those of colica pictonum, that Dr. Young had been led to enquire of him whether he had taken any medicine recently for diarrhea. There could indeed be little doubt, from the account given me of the symptoms, and the subsequent progress of the case, that the attack was one of saturnine colic, of more than ordinary severity.

This attack commenced on the 3d of November, about a fortnight after the patient had taken the last pill, and continued, with occasional slight remissions, for eight days, before any decided relief was obtained. The treatment consisted principally of free and oft-repeated doses of calomel and opium, purgatives by the mouth, enemata and blistering. A very large quantity both of opium and calomel was administered, and every other means employed which an experienced physician could devise, but there was no permanent relief to pain, and no decided action of the bowels, until the eighth day, when the mouth became affected by the mercury. The patient then slowly recovered, but he remained for some time in a very reduced and debilitated condition. It would have been some compensation for all this suffering, if he had been permanently cured of his diarrhea; but no such fortunate result ensued. The attacks continue to recur from time to time, and are as obstinate as ever.

If any should be disposed to doubt whether the above was truly a case of lead colic, in view of the long interval which elapsed between the employment of the remedy and the manifestation of the symptoms, I would remark that this is entirely in accordance with the oft-observed facts in regard to the poisonous operation of lead—*slowness* of action being the general rule.

This is the only case I have ever met with in my own practice, of any serious result following the exhibition of the acetate of lead; indeed, I rarely trifle with the drug now-a-days. ("*Chat echaude craint l'eau froide.*") But I know of three other cases of the same kind, which have occurred in this neighborhood. It is fair to add, however, that in at least two of them the remedy was freely used. There is no lack of recorded instances of the sort; but so far as I am informed, cases are very rare, in which colic is produced by so small a quantity of the acetate as was administered in the case which I have just related. One such is mentioned by Dr. Burton, in a paper of which extracts are given in Braithwaite's Retrospect, No. 2, p. 66; here the patient took fifteen grains in five days, and experienced severe colic. And Prof. Trousseau, in his work on Therapeutics, quotes a case in which the patient took six grains of the neutral acetate of lead daily for three successive days; the fourth day, a most violent saturnine colic supervened, with jaundice, constipation, retraction of the belly, etc., which only yielded to the "treatment of La Charité," energetically employed. The most remarkable case of all is given by Devergie, in his *Med. Legale:* A student of medicine consulted Professor Fouquier, who prescribed for him some pills containing each one grain of acetate of lead, of which he was directed to take one every day. The *first pill* produced slight colic, the second acted more severely, and the third occasioned such violent symptoms that some mistake of the apothecary was suspected. The pills were analysed by Devergie, but were found to contain nothing but acetate of lead.

It may be urged, and with truth, that such cases are exceptional, and that they may be explained by the existence of idiosyncrasies in the subjects of them, such as are known to exist with respect to mercury, opium and other remedies. But allowing this to be true, it must also be admitted that a prudent physician is bound to regard such idiosyncrasies in his practice. The knowledge of their occasional existence should be a warning against the use of hazardous remedies, except under circumstances imperatively calling for their employment.

A circumstance which has doubtless contributed in no small degree to extend the employment of the acetate of lead as a medicine, and to set the minds of physicians at rest in regard to any risk attending it, is the opinion so confidently expressed by Dr. A. T. Thomson, that *the carbonate is the only poisonous preparation of lead*, and that the acetate can only become so in consequence of its decomposition by the free carbonic acid extricated in the alimentary canal. Hence his precept to direct a draught of vinegar to be taken with each dose of the acetate, in order to prevent such decomposition, as well as (I presume) to re-saturate with acetic acid any portion of the carbonate with which the crystals of the acetate may be contaminated.

It requires but little examination of the facts to convince us that this idea is entirely opposed to the well known general laws of the action of mineral poisons. *Ceteris paribus*, these poisons are active in the direct ratio of their solubility; and on this general fact is based the theory of antidotes. We give antidotes with the view of converting a soluble preparation of a metal into an insoluble one. Would it not be a singular exception to a general rule, if the acetate, one of the most soluble forms of lead, should be entirely innocuous, and the carbonate, one of the least soluble, alone endowed with poisonous properties? It is true, that small doses of the carbonate would be rendered soluble by the acids of the gastric juice, being converted by them into the chloride and the acetate, (assuming, in accordance with the general opinion of chemists, that the hydrochloric or acetic acid, or both, exist in the secretion of the stomach.) What then will become of the acetic acetate, when swallowed? If it meets with acid in the stomach, it will remain acetate still; if with hydrochloric acid, it will be be converted, like the carbonate, into the chloride of lead. The two substances, therefore, when they begin to act on the system, and are absorbed into the blood, will be in precisely the same chemical state. Considering the acid nature of the gastric secretion, and its probable action on all chemical compounds capable of being affected by its ingredients, is not Dr. Thomson's idea of the conversion of acetate of lead into carbonate, by the free carbonic reid in the alimentary canal, evidently a fallacious one? And is not therefore his employment of distilled vinegar in conjunction with the acetate, an illusory protection against the dangers of leɛd poisoning?

But without further argument on the chemistry of the question, it is sufficient to state that Dr. Thompson's theory is in opposition to the concurrent testimony of the best toxicological authorities of the day, among whom I may cite Orfila, Apjohn, Taylor, Christison and Devergie. The latter writer, whose authority is second to none, distinctly states that the poisonous activity of the compounds of lead is in direct proportion to their solubility. Christison and Taylor both (conclusively, it seems to me) combat the opinion of Thompson. I will quote a few words from the last mentioned of those authors, bearing directly upon the practical point at issue: "So far as observations on man have yet extended, the carbonate has no more action than the common acetate. Dr. C. G. Mitscherlich has lately proved that the acetate is a poisonous salt, and that when mixed with acetic acid *it is more energetic than when given in the neutral state.* This fact clearly shews that the poisonous effects cannot solely depend on the assumed conversion of the salt to the state of carbonate." [Taylor's Med. Jurisp., 2d ed., p. 169.]

The result of Mitscherlich's researches is precisely what a consideration of the general laws of toxicology would lead us to expect. Let physicians, therefore, take care how they rely on vinegar, or dilute acetic acid, as a *safeguard* against the poisonous effects of the acetate of lead.—*Stethoscope.*

